# Mapping Individual Differences on the Internet: Case Study of the Type 1 Diabetes Community

**DOI:** 10.2196/30756

**Published:** 2021-10-15

**Authors:** Cianna Bedford-Petersen, Sara J Weston

**Affiliations:** 1 Department of Psychology University of Oregon Eugene, OR United States

**Keywords:** type 1 diabetes, diabetes community, social media, Twitter, natural language processing, diabetes community, social network analysis, Latent Dirichlet Allocation, diabetes, data scraping, sentiment analysis

## Abstract

**Background:**

Social media platforms, such as Twitter, are increasingly popular among communities of people with chronic conditions, including those with type 1 diabetes (T1D). There is some evidence that social media confers emotional and health-related benefits to people with T1D, including emotional support and practical information regarding health maintenance. Research on social media has primarily relied on self-reports of web-based behavior and qualitative assessment of web-based content, which can be expensive and time-consuming. Meanwhile, recent advances in natural language processing have allowed for large-scale assessment of social media behavior.

**Objective:**

This study attempts to document the major themes of Twitter posts using a natural language processing method to identify topics of interest in the T1D web-based community. We also seek to map social relations on Twitter as they relate to these topics of interest, to determine whether Twitter users in the T1D community post in “echo chambers,” which reflect their own topics back to them, or whether users typically see a mix of topics on the internet.

**Methods:**

Through Twitter scraping, we gathered a data set of 691,691 tweets from 8557 accounts, spanning a date range from 2008 to 2020, which includes people with T1D, their caregivers, health practitioners, and advocates. Tweet content was analyzed for sentiment and topic, using Latent Dirichlet Allocation. We used social network analysis to examine the degree to which identified topics are siloed within specific groups or disseminated through the broader T1D web-based community.

**Results:**

Tweets were, on average, positive in sentiment. Through topic modeling, we identified 6 broad-bandwidth topics, ranging from clinical to advocacy to daily management to emotional health, which can inform researchers and practitioners interested in the needs of people with T1D. These analyses also replicate prior work using machine learning methods to map social behavior on the internet. We extend these results through social network analysis, indicating that users are likely to see a mix of these topics discussed by the accounts they follow.

**Conclusions:**

Twitter communities are sources of information for people with T1D and members related to that community. Topics identified reveal key concerns of the T1D community and may be useful to practitioners and researchers alike. The methods used are efficient (low cost) while providing researchers with enormous amounts of data. We provide code to facilitate the use of these methods with other populations.

## Introduction

### Background

Social media platforms, such as Twitter, are increasingly popular among communities of people with chronic conditions, including those with type 1 diabetes (T1D) [1]. These platforms potentially have an outsized impact on the daily experiences of people with diabetes, as they provide new opportunities for seeking support, which appears to be a key factor in therapy adherence [2]. Twitter and other platforms also potentially provide instrumental support to people with diabetes and their caregivers through the spread of information regarding new medical treatments or policies (eg, health care). A major challenge to studying the role of social media for people with diabetes is the efficient analysis of content; participants in web-based communities amount to tens of thousands of users, generating millions of posts. This study attempts to document the major themes of Twitter posts using a natural language processing method to identify topics of interest in the T1D web-based community. While this study focuses on people with T1D, we believe similar methods can be employed to explore other health communities on the internet.

### Social Media Benefits People With Diabetes

Prior work documenting the role of social media platforms—including Facebook, Instagram, Reddit, YouTube, Tumblr, and Twitter, as well as community-specific message boards—concludes that social media is mostly beneficial for people with diabetes. This conclusion is primarily based on evidence that the topics of social media posts by the TID community are largely positive and revolve around improving mental health related to diabetes, providing social support, and sharing practical information [3-6]. For example, qualitative analysis of various social media platforms found that posts included themes of humor, pride, and community-building, as well as discussing diabetes-related technology and sharing practical tips [3]. When asked directly, people with diabetes confirm these themes by reporting that web-based communities provide social support, help them feel empowered, and teach practical knowledge for managing diabetes [4,5]. Importantly, concerns about the role of social media platforms in spreading misinformation or negatively impacting self-esteem among young people with diabetes [7] appear to be largely unfounded, as recent synthesis suggests relatively few negative consequences for this community [6].

Subjective impressions about the potential utility of social media are supported by the association of social media with objective indices of health [8,9]. Those who sought health information on the internet were better at testing their blood glucose regularly, taking proper action for hyperglycemia, and adopting nonpharmacological management [8], and bloggers report improved blood glucose levels [9]. In addition to providing emotional support and diabetes-specific health literacy, “diabetes online communities” (DOCs) appear to provide relevant information about navigating health systems [10]. Much of the benefits of DOCs are experienced by not only people with diabetes but also their caregivers [6,11]. It is important to note, however, that these studies rely on observational data; therefore, the causal effect of social media is unknown.

DOCs serve as major sources of advocacy for diabetes communities [7]. In one case, a qualitative assessment of web-based communities suggested that aging individuals are concerned about the limited access to treatment, inability to provide self-care, and health care provider capacity to support aging [12]; the use of web-based platforms brings awareness to these issues and generates the potential for action. For example, Omer [13] documents the “#WeAreNotWaiting” case in which DOCs raised awareness of inaccuracies in glucose monitors, culminating in a web-based chat between patients and the Food and Drug Administration and an in-person meeting to work on these issues.

Finally, social media platforms may benefit people with diabetes by facilitating access to information regarding diabetes. Information may be shared, for example, by health care providers who use social media as a public relations tool [14], to provide advertising services. By sharing information on the internet, health care providers and health researchers have the potential to reduce systematic barriers to accessing new information. In one instance, assessment of social media use around medical conferences suggested that even when only a small proportion of attendees use social media, the information presented at the conference can be widely disseminated to those unable to attend [15,16].

### Scaling Up Social Media Research for Diabetes Communities

As social media websites have gained popularity, the amount of information generated on these sites has increased exponentially. This is a boon to diabetes researchers and presents a methodological challenge: commonly used methods of qualitative data analysis have limited utility in the realm of social media research.

Empirical or data-driven methods of measuring and analyzing social media use can orient research on diabetes communities in several key directions. First, these methods are scalable to large samples of participants. Data-driven approaches forgo the need for interviewers and coders, thus allowing researchers to potentially analyze tens of thousands of participants and millions of posts. Large sample sizes are essential to capturing rare but impactful experiences, which may remain undocumented to this point. For example, while research on DOCs to date has concluded that these communities are supportive and inclusive, it may be that a small subset of individuals experience exclusion or bullying on the internet. Small samples may not capture these individuals, or only include a few of them, thus failing to identify these experiences. In addition, large data sets allow researchers to explore the role of social media in the experiences of caregivers, clinicians, policy advocates, and others invested in the diabetes community and interactions within and across roles.

Second, data-driven methods allow rapid assessment of events or changes, preparing researchers and clinicians for faster response. For example, the political debates around universal health care or changes in national health insurance coverage are important concerns to people with diabetes, as these changes often impact the price and availability of insulin and glucose-monitoring technologies (eg, the #WeAreNotWaiting advocacy and awareness campaign [13]). Researchers can analyze the response to such debates in real-time using models which take a data-driven approach.

### Identifying Topics of Discussion

A challenge with data-driven approaches to analyze large data sets is that many techniques work in a “black box,” obscuring relationships between variables and making the interpretation of statistical models difficult or impossible. For example, many machine learning models that are used to assess large pools of data primarily prioritize out-of-sample prediction rather than interpretable synthesis [17]. Recent advances in linguistic analyses pave the way for empirical analyses of web-based behavior and allow for the synthesis of web-based behavior, thus leveraging large data sets while maintaining focus on descriptive models, rather than predictive models.

Latent Dirichlet Allocation (LDA) [18,19] is one such method for summarizing web-based behavior. LDA is a topic modeling technique that seeks to identify underlying themes that can be used to classify text in a document (eg, a user’s set of tweets). This analysis attempts to uncover a hidden process without input or assumptions from researchers as to the primary themes of the documents. Importantly, LDA allows for mixed membership or for a single document to contain 2 or more topics. LDA analysis has already been successfully applied to social media: tweet (Twitter posts) topics are associated with county-level obesity rates [20] and predict individuals’ risk of developing chronic health conditions [21].

Other machine learning–type methods have also been used to analyze web-based behavior. Relevant to this study, Ahne et al [22] identified tweets related to diabetes through the use of keywords and hashtags and summarized the topics therein using *k*-means clustering. They identified a set of 30 topics, several of which were variations on concerns regarding insulin pricing and availability. These results are promising, in that the majority of topics identified were easily understood by researchers and clearly connected to major concerns of people with T1D. However, inclusion of only diabetes-related tweets—rather than all tweets by people with T1D—potentially omits important experiences by these communities. Moreover, it is unclear how these topics are transmitted within DOCs. For example, is insulin pricing a topic discussed in detail by a subset of accounts or disseminated broadly throughout the community? With these questions in mind, we turn to the current study.

### The Current Study

This study seeks to empirically assess the use of Twitter by the T1D community, including persons with diabetes, caregivers, medical professionals, advocates, and policy makers. We aim to address 3 primary research questions: (1) what is the overall sentiment of social media posts? (2) What are the major topics of discussion on the internet? (3) How is the social network of Twitter users organized around topics of discussion?

Of note, similar analyses of Twitter use by people with (all types of) diabetes were conducted recently by Ahne et al [22]. While our study is both conceptually and analytically similar to that of Ahne et al [22], we expand on the methodology and research questions in two ways: first, data collection was driven by the goal of including members of the type 1 DOC, rather than tweets covering a specific topic. This allows us to generate a more holistic view of these users’ lives and concerns. Second, by including network analyses, we can investigate how topics are being shared within DOCs, whether users are exposed to a large number of topics or a narrow subset, and to what extent there is a single large DOC or many smaller ones on Twitter.

## Methods

### Sample and Data Collection

To begin identifying tweets in the T1D community, we used the following hashtags: #t1d, #t1dlookslikeme, #brokenpancreas, #type1kid, #typeonetypenone, #diabadass, #type1warrior, #beyondtype1, #insulindependent, #typeonestrong, #dexcom, and #GBdoc. This list was generated through discussion with Twitter users within the T1D community and an informal survey of tweets. We avoided using more generic hashtags such as #diabetes, which may also include tweets from those in the type 2 diabetes community, which were not the focus of this study. Using the Rtweet package (version 0.7.0) [23] in R, we pulled 1500 tweets containing these hashtags over the prior week (December 28, 2019, to January 3, 2020). These tweets represent a mixture of the most recent tweets and the most popular tweets during that 1-week period.

**Figure 1:**
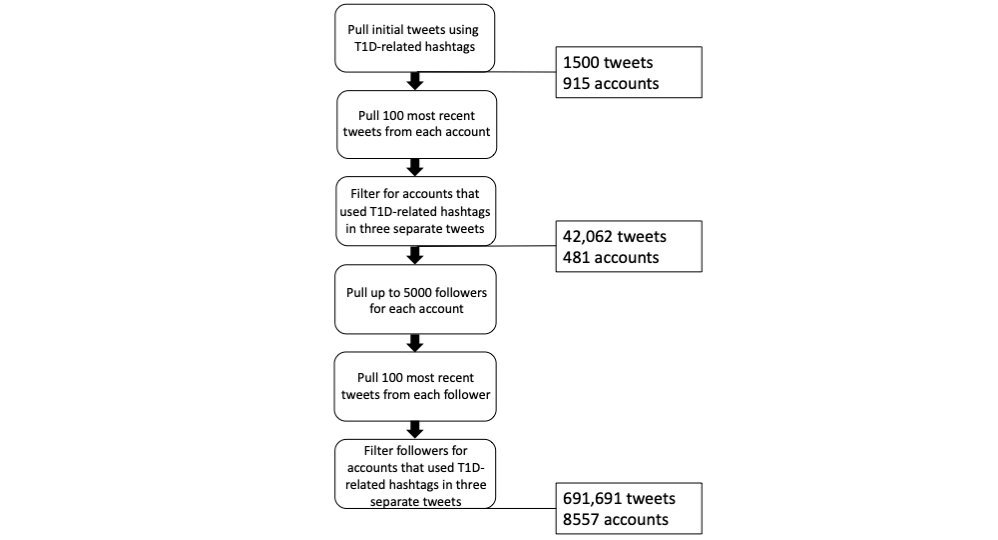
Tweet collection procedure. T1D: type 1 diabetes.

In this initial pull, we gathered 915 unique Twitter accounts. In line with our goal to include all tweets from T1D community members, not just tweets about T1D, we pulled the 100 most recent tweets (including retweets and replies) from each of these accounts. Additionally, to make sure that the accounts we pulled were accounts with T1D as a recurring topic of tweets, we included only accounts with at least 3 separate tweets containing at least 1 of the T1D hashtags (481 accounts and 42,062 tweets). Finally, we recognize that not all people with T1D will have tweeted about their diagnoses within the past week. However, these individuals are more likely to follow accounts that include frequent posts about T1D. Therefore, we attempt to capture more members of the T1D web-based community by pulling the Twitter followers of the accounts in our data (up to 5000 followers for each account). For each of these followers, we pulled 100 of their most recent tweets. We again included only those accounts where there were 3 separate tweets containing any of our selected T1D hashtags, to restrict the accounts included to those in the T1D community. Finally, for consistency in our natural language processing results, we included only those tweets written in English. Our final analysis sample consisted of 691,691 tweets from 8557 accounts (Figure 1).

Tweets used in this analysis spanned a date range of April 4, 2008, to January 15, 2020. Just over half (54%) of the tweets in our sample occurred after January 2019, within approximately 1 year of our data collection date, and 69% of tweets occurred within 2 years of our collection date.

### Data Analyses

Prior to analyzing our tweets, URLs were removed from our sample of tweets as well as greater-than signs (>), less-than signs (<), ampersands (&), and the letters “RT,” which denote the classic version of the retweet. These characters were removed because they do not contribute to the sentiment of a tweet and are often not handled well by language processing methods [24]. Finally, we removed the set of hashtags initially used to search for and identify T1D tweets as they are oversampled in our set of tweets.

To address our first research question (ie, “What is the overall sentiment of social media posts?”), we analyzed our tweet sample using sentiment analysis. This approach, also known as opinion mining, is used to determine whether a given text is positive, negative, or neutral. For this study, we are interested in, on average, how positive or negative a user’s set of tweets is. We can accomplish this using the Noncommercial Research (NRC) sentiment lexicon [25], a sentiment dictionary designed for and validated with tweets; this includes a large set of words where each word has been assigned a score for positive/negative sentiment (ranging from –6.93 to 7.53). This set of words is then compared to the words in a user’s tweets, giving us an average sentiment for each user. Finally, we are able to take an average of sentiment across all our users to get a sense of overall sentiment in our T1D web-based community.

Next, we answered our second research question (ie, “What are the major topics of discussion on the internet?”) using the natural language processing technique of LDA [18], an unsupervised machine learning algorithm that identifies latent topic information among large document collections. Unlike other topic modeling methods, LDA does not focus on the frequency of words but rather assumes that a topic is made up of a probability distribution of words. A topic is a list of words. Each word is assigned a probability value for each topic, which represents the likelihood that the word would be used in a document containing that topic. LDA assigns to each document latent topics together with a probability value that each topic contributes to the overall document. In this case, a document refers to a user’s 100 most recent tweets.

**Figure 2:**
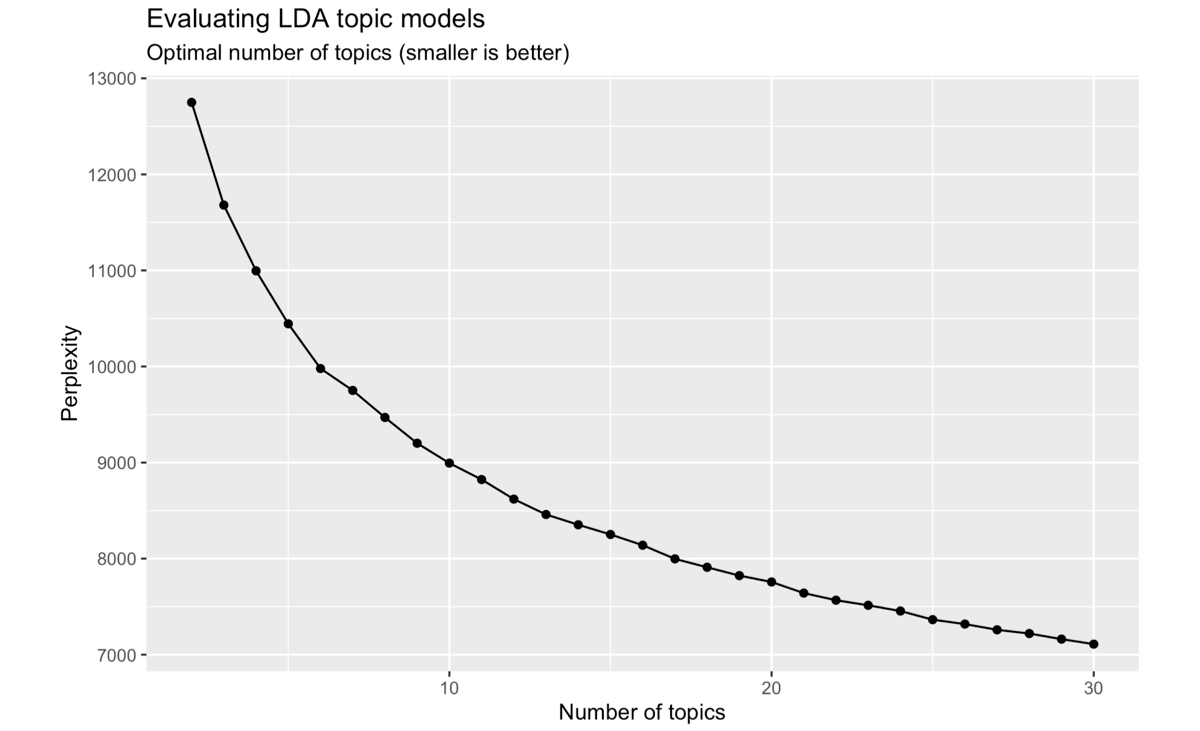
Perplexity by the number of topics in Latent Dirichlet Allocation models. LDA: Latent Dirichlet Allocation.

Similar to other data reduction methods (eg, factor analysis), researchers must choose the number of latent topics to fit. We used both perplexity (a quantitative index) and subjective interpretability to decide how many topics to fit. *Perplexity* measures how poorly a probability model predicts a sample. More specifically, the normalized log-likelihood of a held-out test set of data is used to determine how “surprising” the test set is, considering the model. We fit many LDA models, each for a different number of topics (Figure 2) and calculated the perplexity score for each. Per usual, an LDA solution with more topics results in lower perplexity, which indicates superior prediction in our model. While lower perplexity is desirable, interpretability of the latent topics is also important. While a 30-topic model appears ideal in terms of predictive utility, this large number of topics was difficult to interpret (Multimedia Appendix 1 shows the 30-topic model). We instead chose 6 topics as our final model, which appeared to be a sort of elbow in our perplexity chart and showed generally interpretable topics. For sensitivity analyses, we fit LDA models with 5, 7, and 8 topics, and the latent topic categories appeared very similar.

For our third and final research question (ie, “How is the social network of Twitter users organized around topics of discussion?”), we used social network analysis [26]. Here, we mapped a network of the top followed accounts in our tweet sample, connecting accounts on the basis of whether one follows the other. We colored nodes (accounts) on the basis of the dominant topic in their tweets. Unlike the previous 2 analyses, this method is a more qualitative representation of data. Interpretation of a graphical display of the social network—in which individual Twitter users, or “nodes”—are color-coded in accordance with their most common topic is somewhat subjective. Similar methods have been used in other research to map comments related to Japanese and Korean public diplomacy organizations [27], as well as contributions of websites related to the food safety movement in the United States [28]. Together, these methods provide an insight into how the community connects and interacts.

All analyses were preregistered on the Open Science Framework [29]. Twitter prohibits the sharing of tweet content, but we are allowed to share tweet IDs and user IDs for the tweets analyzed here. That data file, as well as all R code for these analyses, can be found on the Open Science Framework [30]. Interested researchers can use these data to identify the tweet content using the Twitter application programming interface.

## Results

### What is the Overall Sentiment of Social Media Posts?

**Figure 3:**
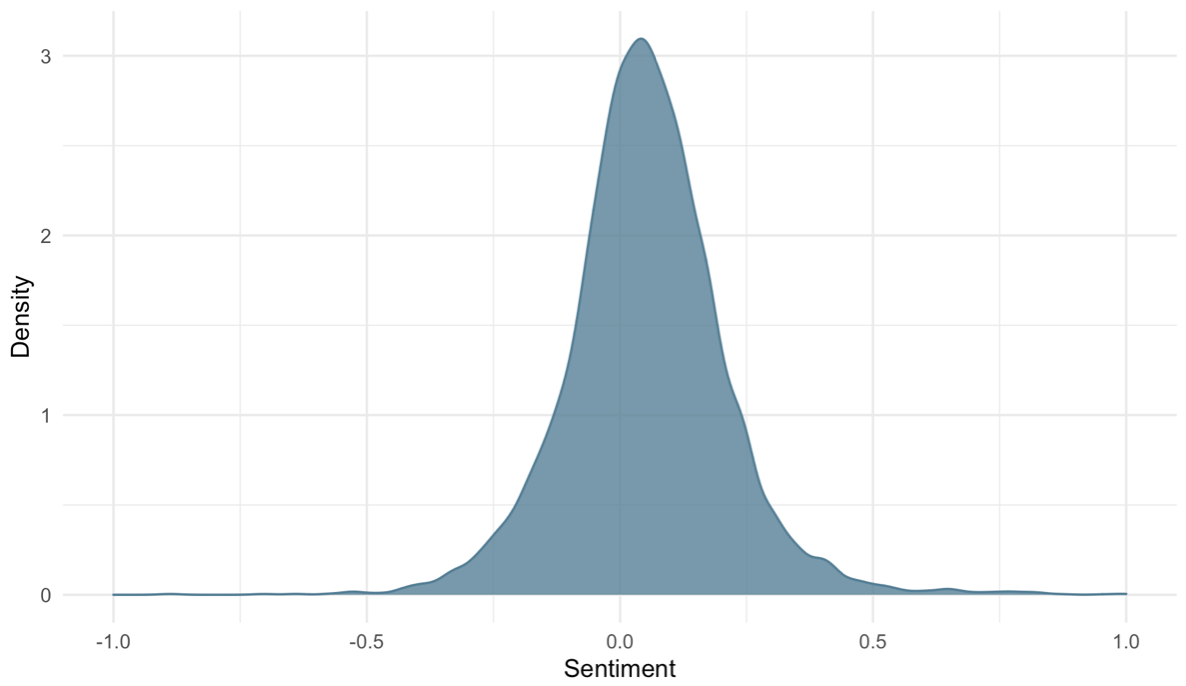
Distribution of sentiment for our type 1 diabetes tweet sample.

The NRC sentiment lexicon [25] was used to answer our first question regarding the overall sentiment in our sample of Twitter posts. The sentiment score of a user is the average of the sentiment score of their words across all tweets. As such, user sentiment is independent of the number of times the post or the length of their posts. User sentiment ranged from –2.03 to 1.64, with an average score of 0.052 (Cohen *d*=0.32), indicating an overall slightly positive sentiment of user tweets (Figure 3). Within our sample, 64% of users had a sentiment that was greater than zero, indicating that the sentiment of their tweets was more often positive than negative.

### What are the Major Topics of Discussion on the Internet?

**Figure 4:**
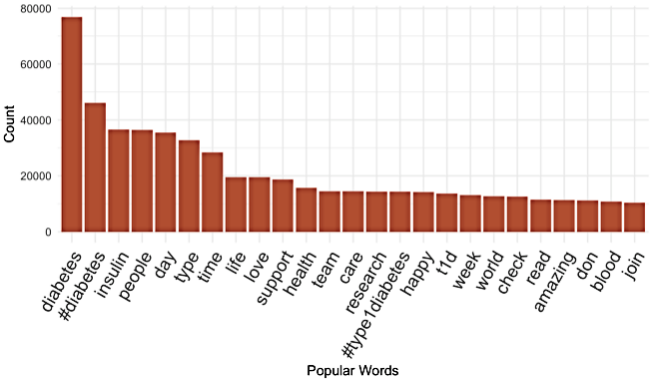
Most popular words in our type 1 diabetes tweet sample.

Prior to running the LDA analysis, we first looked at the most popular words in our sample (Figure 4). Top words included very explicit indicators of diabetes and related management tools including diabetes, #diabetes, and insulin. While these words are not surprising to see, they serve as an indicator that our method of pulling tweets accessed the community we were targeting. Additionally, we noted a strong theme of encouragement with popular words of love, support, and care.

**Figure 5:**
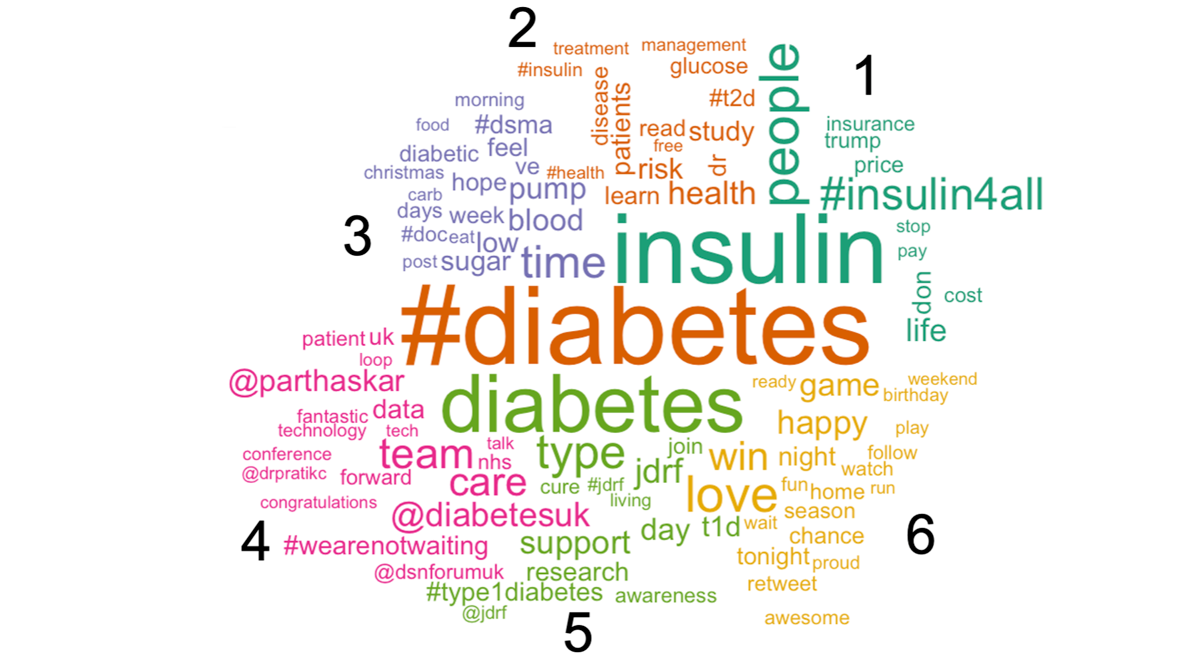
Comparison cloud of the most likely words to appear in each topic of our 6-topic Latent Dirichlet Allocation model.

Next, we extracted 6 topics using the LDA approach. To ensure sufficient document length, we aggregated tweets within accounts to create a single document. This allows us to characterize the content generated by each user, but we are unable to disaggregate these results to individual tweets. After extracting topics, we examined the words most likely to appear in each topic using a comparison cloud (Figure 5). Thereafter, we examined tweets from users, which had the highest probability of being assigned that topic to gain context for the most likely words and help generate descriptions for each topic. Topic 1 was centered around the *insulin price crisis*, which refers to the drastic increase in insulin prices since the 1990s and the call for access to affordable insulin as a human right. This topic additionally references Donald Trump and his involvement with this movement. The *insulin price crisis* accounted for approximately 19% of words across all tweets. The second topic is about T1D *clinical research* including reference to studies, risk, patients, and treatment. This is focused on new developments in the clinical trials area of research, and accounted for 14% of words. Topic 3 addressed *daily management of T1D* and featured tools including a pump as well as eating-related words including “sugar” and “carb.” This topic was the most prevalent, accounting for 23% of words. The fourth topic in our model highlighted *technology advancements* using words including “loop,” referring to the concept of a closed-loop system or “artificial pancreas.” This method of T1D blood sugar regulation combines a continuous glucose monitor and an insulin pump to manage insulin delivery with minimal interaction required from the patient. This topic also heavily utilized the hashtag #wearenotwaiting, referencing a movement of those in the T1D community who are taking technology development into their own hand with new apps and cloud-based solutions that utilize patient health data to inform blood sugar management. However, this topic was also among the least prevalent, accounting for only 13% of words. Topic 5 encompasses the many *awareness organizations* that utilize Twitter to educate the public about T1D and related fundraising events. This topic accounted for 13% of words tweeted. Finally, the sixth topic seems to encompass *positive emotions* with words including “love” and “happy” as well as life outside of T1D using words such as “watch,” “run,” “game,” “home,” and “weekend,” and this topic accounted for 18% of words. This topic is notable, especially given the goal of studying all tweets from the T1D community and not only those tweets specifically about diabetes. Taken together, these topics give us a broad view of the key topics discussed on the internet in the T1D community (Table 1). We looked at the relationship among our 6 topics by correlating the probability of a user’s tweets being in a given topic. Correlations, were negative, ranging from –0.12 to –0.26. Low magnitude suggests that topics are relatively distinct (ie, not highly associated), but also that as accounts include more content related to one topic, they are less likely to include content related to the others.

**Table 1:** Example tweet for each latent topic.

Latent topic	Example tweet
Insulin price crisis	“It’s not my fault that insulin costs so much. I’m doing my part as a citizen, I’m working. I have these benefits. I can get my teeth cleaned, my eyes checked, but I can’t get the medicine that keeps me and my sister alive. #insulin4all”
Clinical research	“@WNDU reports on @T1D_TrialNet's groundbreaking study that showed an immunotherapy drug delayed a #type1diabetes diagnosis by two years. #immunotherapy”
Daily management of T1D	“My Monday and Tuesday blood sugars were BEAUTIFUL Today my blood sugars were garbage bc I should have changed my site earlier And I’m okay with that. Here’s your reminder to pat yourself on the back for the good days, and learn from your mistakes on the bad ones!”
Technology advancement	“Managing my sons bs while he sleeps in the USA while on the Amalfi coast! I’m #forevergrateful to you all @NightscoutFound @WeAreNotWaiting #wearenotwaiting #tripofalifetime #sohardtoleavehim”
Awareness organizations	“Walk with us to turn Type One into Type None. By donating or registering today, you will help JDRF create a world without Type 1 Diabetes (T1D)”
Positive emotions	“Had such a great and full weekend. Went for a drive to the Gold Coast after training on Saturday and ate some great vegan food from Govindas then went for a long walk on the beach to reflect on the past week”

### How is the Social Network of Twitter Users Organized Around Topics of Discussion?

**Figure 6:**
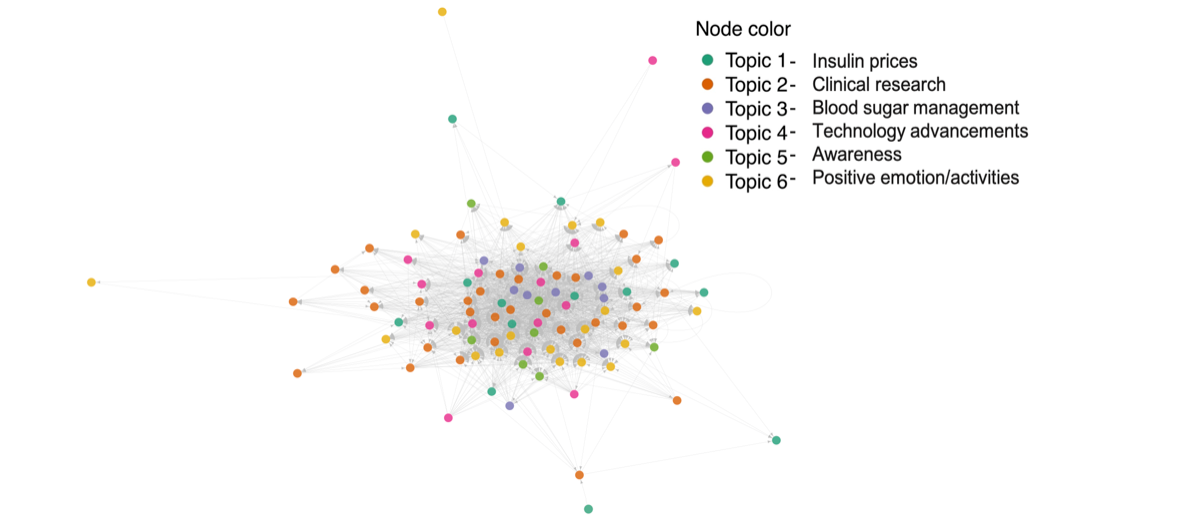
Social network analysis of the top 100 most followed accounts in our type 1 diabetes tweet sample. Nodes represent Twitter accounts and edges represent follows. The color of each node represents the dominant topic of each account in correspondence with the 6-topic LDA model.

To address how the social network of Twitter users organized around topics of discussion, we used social network analysis (Figure 6). While it would be ideal to complete this analysis with all 8557 participants in our sample, this would not be feasible with the personal computing power available to us. Instead, we narrowed our sample to the 100 accounts with the most followers. This provided us with a sample of highly influential accounts within the T1D web-based community for assessment. These accounts ranged from having 7202 followers to 278,180 followers and spanned a wide range of identities including research or awareness organizations, public figures including actors or singers, blog- or community-focused accounts, and doctors. In our social network analysis, each node represented a Twitter account, and each edge represented a follow. The color of each node represents the dominant topic of each account in correspondence with the 6-topic LDA model described above. The dominant topic was determined by selecting the topic with the largest per-document-per-topic probability; that is, the probability of each topic within each account’s set of tweets.

Our analysis showed that there is a considerable amount of intermingling among dominant topics in our group of influential Twitter accounts. One possibility is that we would see distinct clusters of colors in our analysis, indicating that groups were primarily following accounts that had the same dominant topic as their own account. Instead, we see considerable overlap in dominant colors across our network of accounts. This indicates that influential accounts in the T1D web-based community see a wide range of topics on their Twitter feed rather than just the topic that dominates their tweets. It was observed that there is a cluster of topic 2 (*clinical research*), which accounts at the center of our network, indicating that these accounts are the most followed within the T1D community. Accounts with *positive emotions* as their dominant topic rarely appear at the center of our network. While these accounts do appear to follow other accounts in the network, they appear to be somewhat less integrated. This may be an indication that, while they may be members to the T1D web-based community, TID may not be central to their web-based identity. Accounts primarily tweeting about *insulin prices* also tended to hang around the edge of our network, and those accounts were followed by very few others within our network. The insulin price crisis affects those beyond the T1D community and is also frequently discussed by politicians or those who work in policy- or insurance-related fields. Finally, we observed that within our sample of 100 top followed accounts, *clinical research* was the most common dominant topic (34 accounts). This was followed by *positive emotions* (21), *technology advancement* (14), *insulin price* (13), *daily management of T1D* (10), and finally *organization* (8). In contrast, management of T1D was the most popular topic in our full sample of 8557 accounts.

## Discussion

### Principal Findings

The current study examined the tweets and network structure of accounts within the T1D Twitter community, demonstrating the feasibility of latent topic modeling as a tool to analyze the use of social media by this and other communities. We identified several broad-bandwidth topics, ranging from clinical to advocacy to daily management to emotional health, which can inform researchers and practitioners interested in the needs of people with T1D. Moreover, network analysis suggests that users are likely to see a mix of these topics discussed by accounts they follow.

Importantly, these findings converge with prior conclusions regarding web-based engagement, such as those web-based communities serving as sources of positive emotion [3], providing practical support [3-5], advocating for needed health care reforms [12,13], and disseminating results from clinical research [15,16]. Compared to prior work, however, these analyses incorporated a very large number of users and made use of algorithmic methods to categorize web-based messages. Despite using different methodology to select tweets for inclusion and for identifying major topics of interest, we replicate recent work by Ahne et al [22], who reported that a major concern of the Twitter DOC is insulin pricing. We also recovered several other major topics, such as diabetes awareness and support, and our *positive emotions* topic may correspond to “enjoying the exchange in the diabetes online community” [22], although the content of the positive tweets in our data appeared more tangential to diabetes. However, a major divergence between these projects was the choice of number of topics to extract and evaluate (6 in ours, compared to 30). A greater number of topics provides the benefit of specificity and nuance, although there is also greater susceptibility to trends, niche topics, and coincidences. For example, Ahne et al [22] found among their 30 topics a discussion of the pop star Nick Jonas (who has been diagnosed with T1D) and advertisements for a makeup product called Bloodsugar. It is unclear as to whether topics such as these are irrelevant to the research goals of psychologists and clinicians or whether they represent sources of advocacy and normalization. Judgement may be made depending on the specific topics extracted and the goals of a particular analysis. Certainly, niche and trend topics inform the understanding of cultural influences and inner lives of people with diabetes, but they may have limited predictive power for broad outcomes. Speaking more broadly, fewer numbers of topics may be more generalizable and easier to track over time, although they lose specificity. Differing numbers of topics are likely useful for different research questions. For example, future research might include pairing Twitter information with real-world outcomes (eg, HbA1c levels) to identify the topics that predict changes in health status.

We believe the current research demonstrates the utility of the LDA method for utilizing social media data in studies on type I diabetes and for patients with chronic illnesses more broadly. Indeed, these analyses could be easily applied to other communities by simply changing the initial key words and hashtag search. Through open-source software, we were able to analyze nearly 700,000 tweets from more than 8000 accounts. Given the feasibility of these analyses, we anticipate they could be used for a number of purposes. Simple adaptations of our code will allow for the study of other communities of people with chronic conditions (eg, cancer survivors or autoimmune conditions). Alternatively, linking Twitter with other forms of data collection (eg, self-report or biological assessments) can be used to study the association between social media engagement and real-world outcomes.

### Limitations

However, these methods are not without their limitations. In contrast to more recently developed natural language processing methods, LDA is not based on word embeddings and does not take sentence structure into account as it assumes that words are exchangeable. It also cannot be argued that Twitter users are representative of the United States or world populations, nor do we expect them to be representative of all people with T1D. We expect to have undersampled older adults [31] and communities with limited or unreliable internet access, and there are expected issues with sampling related to geography and race/ethnicity [32]. Notably, however, Black people may be more highly represented on Twitter (compared to other ethnic groups) [33], creating an advantage to using this platform in that researchers can reach populations typically underserved. More specific to this population, our method of selecting participants in the study on the basis of the content of their most recent 100 tweets will not capture Twitter users who choose not to disclose their T1D status on the internet. This exclusion is arguably not relevant to the research question, “What is the focus and network structure of diabetes online communities?” as these users would not participate in these communities.

### Conclusions

In sum, the current study contributes to a growing literature of examining the use of social media by people with chronic conditions; in this case, T1D. These findings show that health researchers can leverage the vast amount of data available on Twitter (and potentially other platforms) to efficiently understand major concerns of these populations. Moreover, these findings support prior work showing that people with chronic conditions may use social media to access practical information and social support.

## Appendix

Multimedia Appendix 1 Comparison clouds of the most likely words to appear in the 30-topic Latent Dirichlet Allocation model.

## Abbreviations

DOC: diabetes online community
LDA: Latent Dirichlet Allocation
NRC: Noncommercial Research
T1D: type 1 diabetes
