# Supplementary material for: Mapping Individual Differences on the Internet: Case Study of the Type 1 Diabetes Community
Source: JMIR Diabetes. 2021 Oct 15;6(4):e30756. doi: 10.2196/30756 (PMC8556640; doi:10.2196/30756)
Supplement: Multimedia Appendix 1 [file diabetes_v6i4e30756_app1.docx]

**Multimedia Appendix 1.** Comparison clouds of the most likely words to appear in the 30-topic Latent Dirichlet Allocation model.

Comparison cloud of the most likely words to appear in topics 1-6 of our thirty topic LDA model.


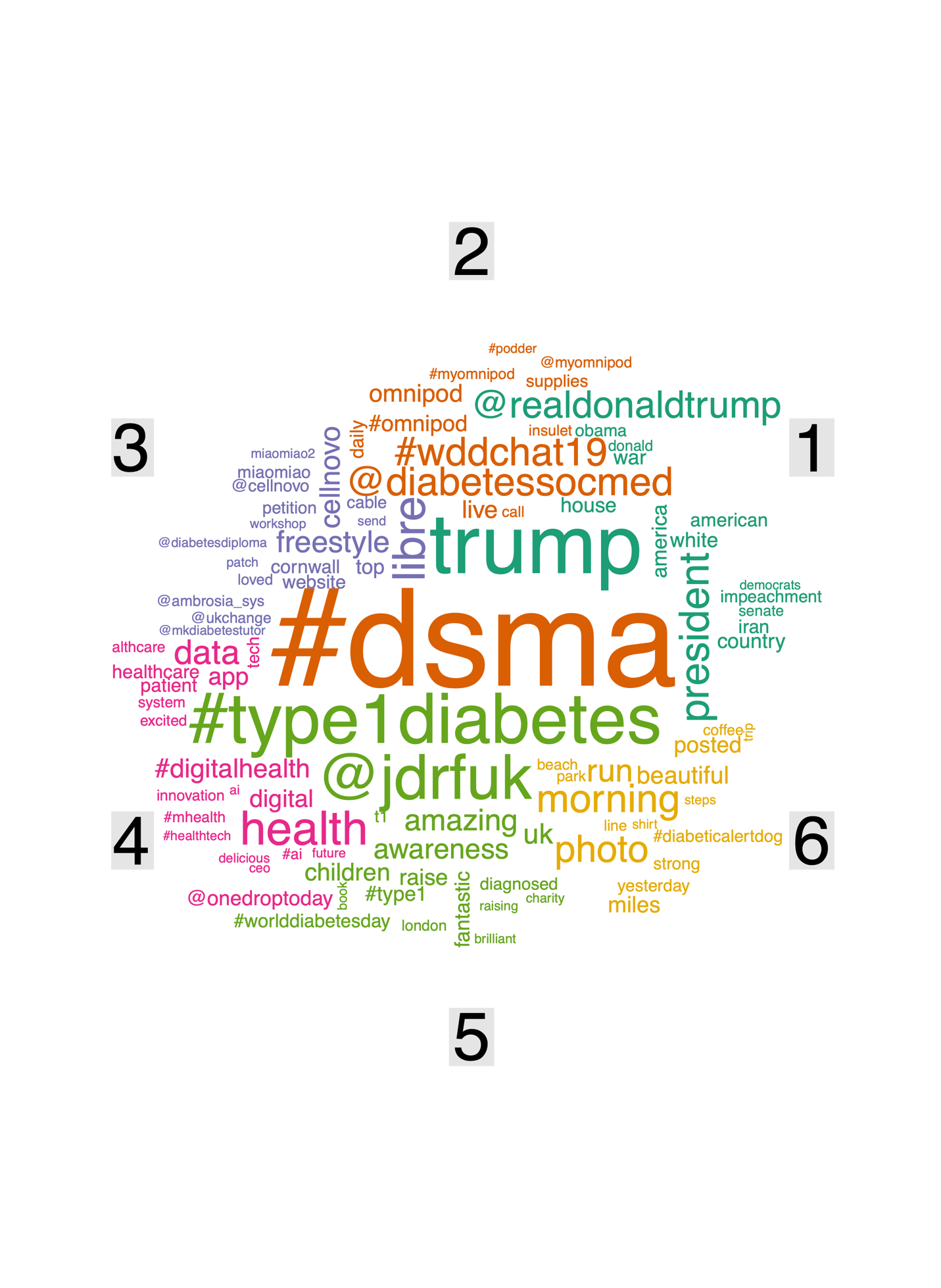


Topic 1: American politics

Topic 2: Omnipod

Topic 3: Glucose management technology brands

Topic 4: Digital health

Topic 5: Diabetes awareness organizations

Topic 6: Activity (difficult to interpret)

Comparison cloud of the most likely words to appear in topics 7-12 of our thirty topic LDA model.


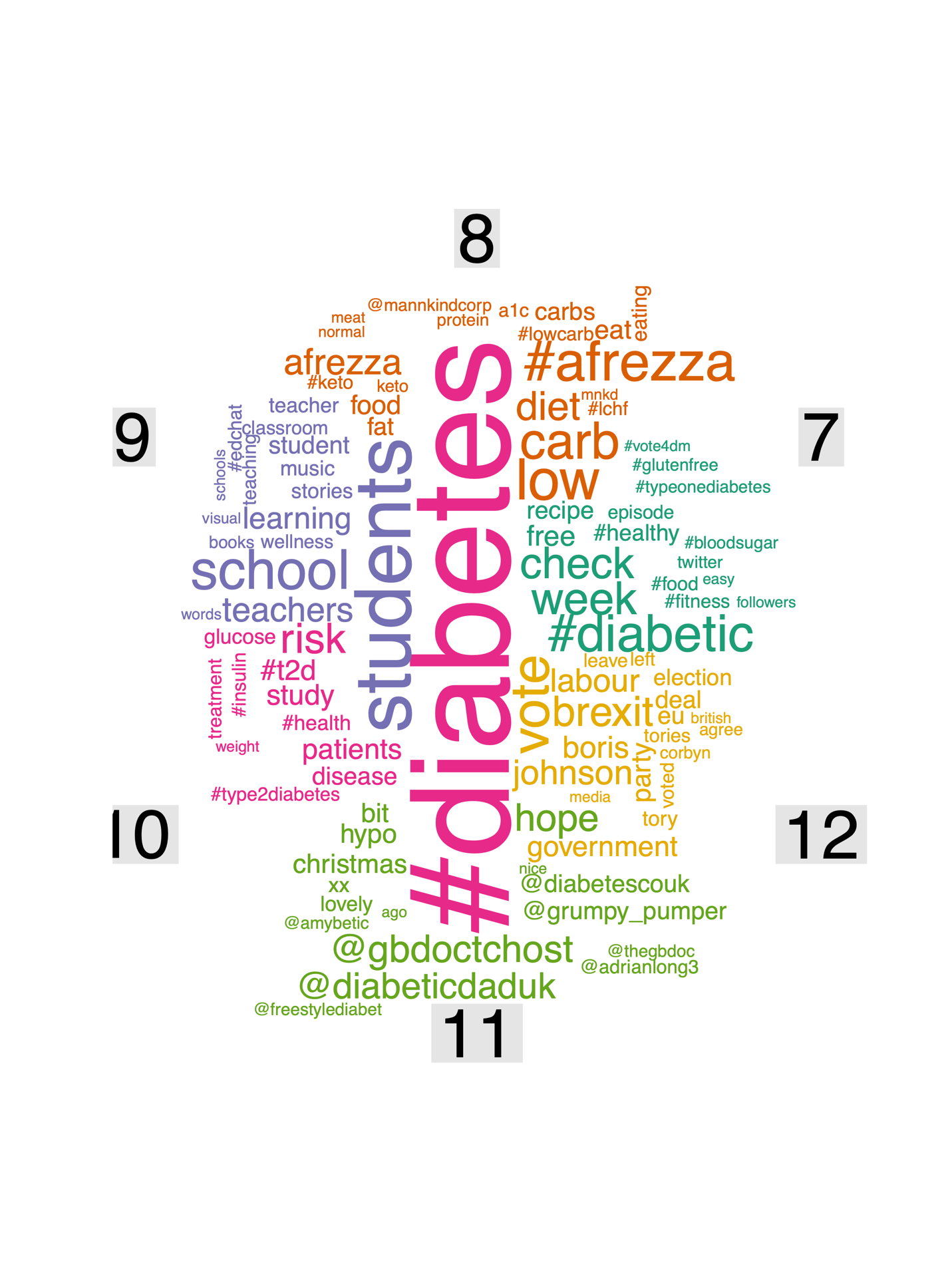


Topic 7: Cooking (difficult to identify)

Topic 8 Food (low carb)

Topic 9: Teaching

Topic 10: General diabetes, Type 2

Topic 11: Unable to identify

Topic 12: British politics

Comparison cloud of the most likely words to appear in topics 13-18 of our thirty topic LDA model.


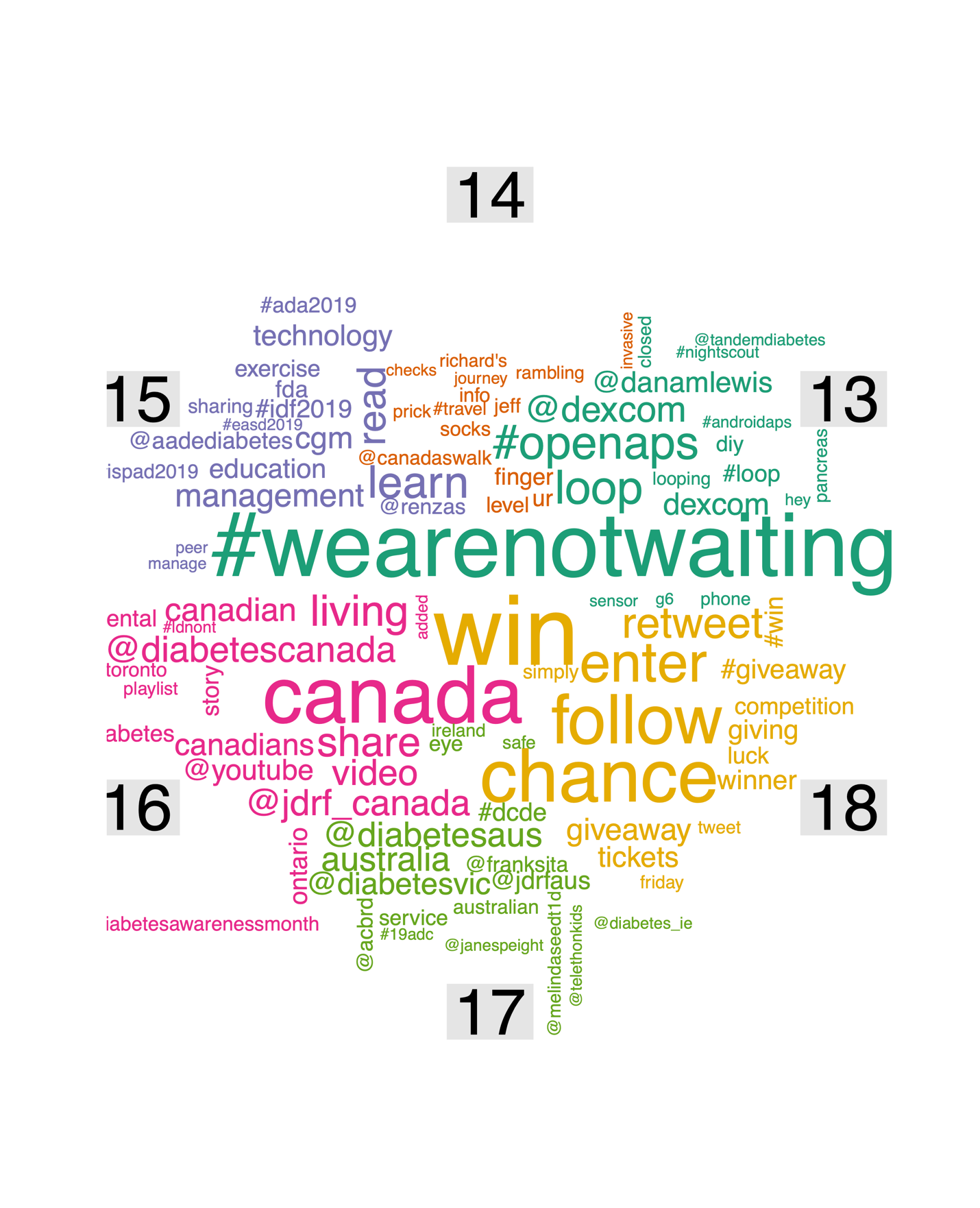


Topic 13: Closed loop technology

Topic 14: Unable to identify

Topic 15: Glucose management technology (general)

Topic 16: Canada

Topic 17: Australia

Topic 18: Giveaways

Comparison cloud of the most likely words to appear in topics 19-24 of our thirty topic LDA model.


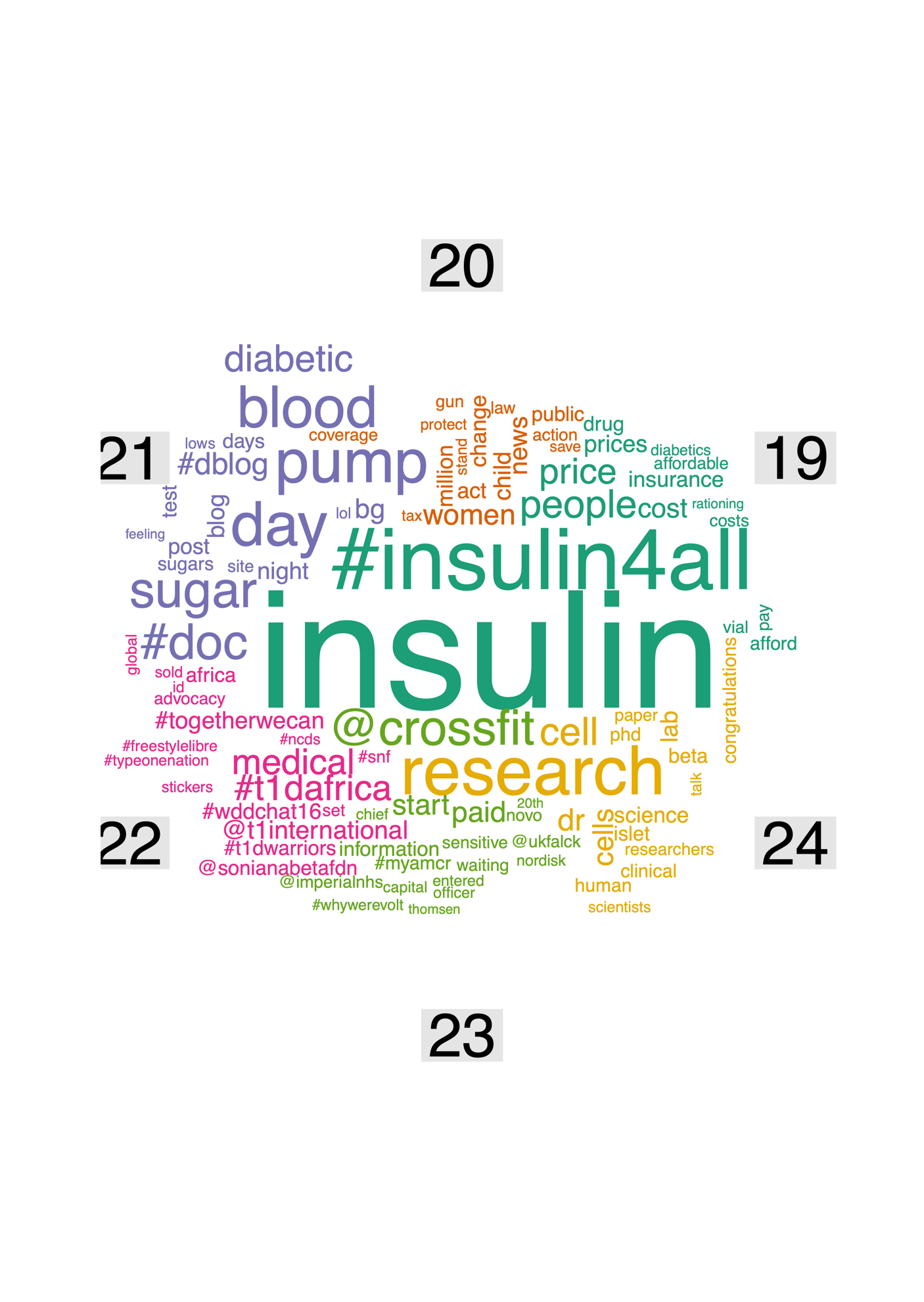


Topic 19: Insulin price crisis

Topic 20: Unable to identify

Topic 21: Blog/General diabetes management

Topic 22: International diabetes community connection

Topic 23: Unable to identify

Topic 24: Cellular research

Comparison cloud of the most likely words to appear in topics 25-30 of our thirty topic LDA model.


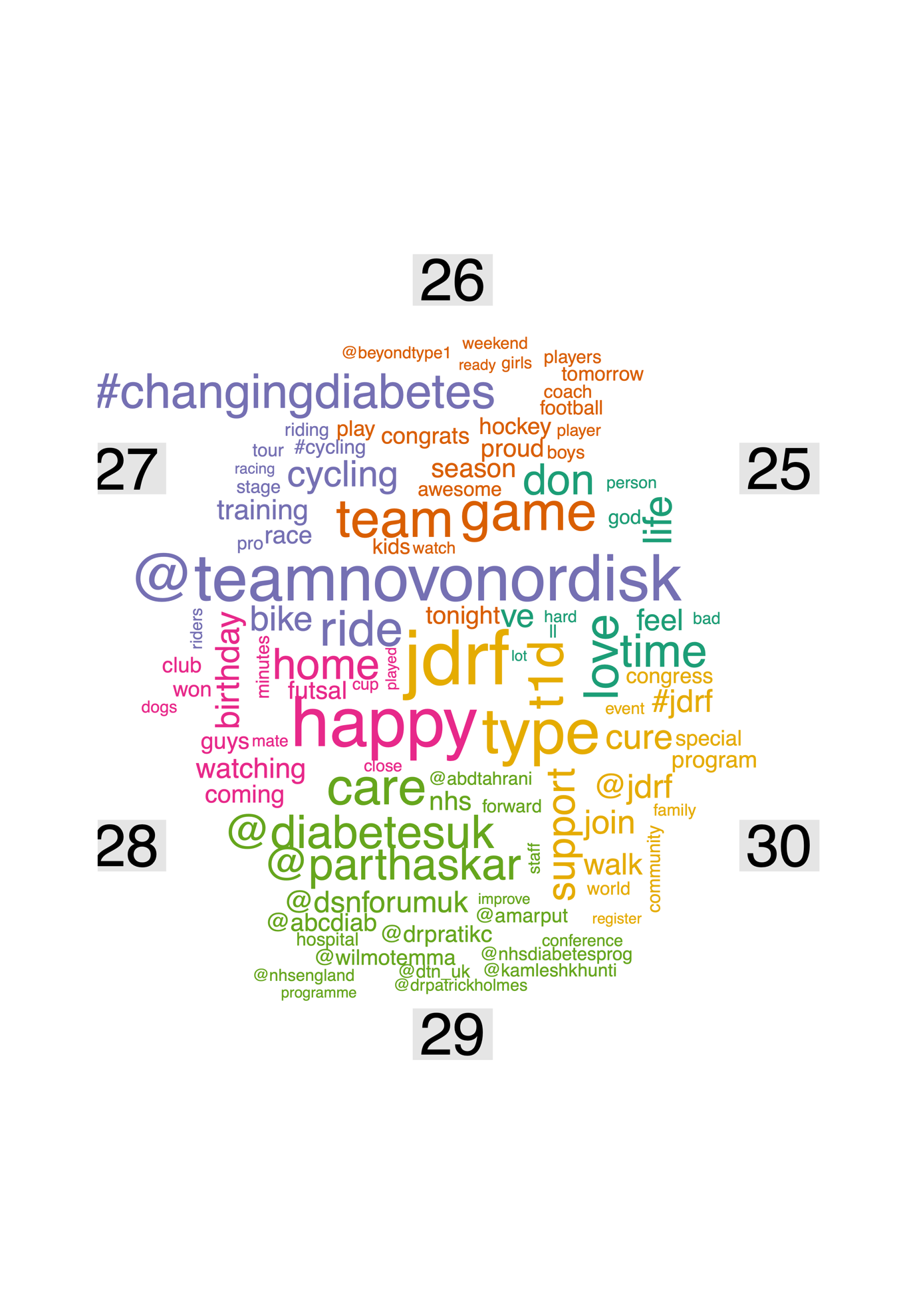


Topic 25: Emotion words (difficult to identify)

Topic 26: Sports

Topic 27: Cycling

Topic 28: Unable to interpret

Topic 29: Prominent account names in the DOC

Topic 30: Diabetes awareness organizations and events
